# How surgical rotations in medical school contribute to students’ perceptions and aspirations of a career in surgery: A qualitative study

**DOI:** 10.1016/j.jpra.2025.09.021

**Published:** 2025-09-25

**Authors:** Eloise Owen, Jack Bennett, Chetan Parmar, Sjaak Pouwels

**Affiliations:** a Department of Surgery & Cancer, Section of Vascular Surgery, Faculty of Medicine, Imperial College London, United Kingdom; b Department of Diabetes and Endocrinology, Liverpool University Hospitals NHS Foundation Trust, Liverpool, United Kingdom; c Department of Surgery, Whittington Hospital, London, United Kingdom; d University College London, London, UK; e Apollo Hospitals Educational and Research Foundation, India; f Department of Surgery, Bielefeld University—Campus Detmold, Klinikum Lippe, Detmold, NRW, Germany; g Department of Intensive Care Medicine, Elisabeth-Tweesteden Hospital Tilburg, the Netherlands

**Keywords:** Surgical rotations, Career planning, Student experience, Surgical learning, Medical education

## Introduction

Medical school is a pivotal time whereby students will be exposed to different specialties. Spending time within these specialties and what experience they have there can shape and formulate their ideas about which future career path they will take.^1^^,^^2^ Medical students have their first insight of a career in surgery throughout their time at university. This is usually through a placement or rotation in a surgical specialty. Typically, students spent time in a variety of departments such as general surgery, otolaryngology, vascular, cardiothoracic and breast surgery. The length of time spent in a specialty can differ, but students typically get the opportunity to observe in theatre, learn about pre and post-surgical care, attend clinics, and interact with patients.^3^, ^4^, ^5^

The aim of this qualitative study was to understand the factors, which influence student’s perspectives regarding a career in surgery. One of the study goals was to ascertain whether student’s surgical rotations have had a positive or negative impact of a career in surgery, investigating the unique push and pull factors which contribute towards the student experience and subsequently students’ aspirations.

### Methodology

To learn about the experiences of students on their surgical placements, a *Google Form* online survey was designed. The survey was assembled and edited by all authors. The inclusion criteria were that the participants must have completed a surgical rotation or block (for at least 1 month) as part of their university course, and they must be enrolled and currently studying medicine. The questionnaire consisted of seven questions, with open-ended free text responses, with an unlimited number of words. The software NVivo was used to assist all the qualitative analysis in this study.

## Results

Twenty-six students from seven different countries responded to the survey. One student who completed the survey did not match the inclusion criteria because they had not undertaken a surgical placement, so their results were excluded. Students who completed our study had undertaken placements in a range of surgical specialties, including general surgery, orthopedics, vascular, ENT, breast, cardio-thoracic, and plastic surgery.

### Demographics

**Figure 1:**
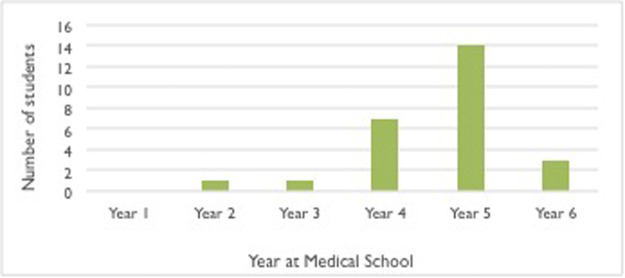
Graph to show what year of study the medical students were who completed the survey.

Majority of students who completed this study were in the latter half of medical school, as the graph below shows (Figure 1). This survey was completed from students across a range of countries, but predominantly the United Kingdom (Table 1).

**Table 1:** Locations of the countries where the students attended medical school.

**Country**	**Number of students**
United Kingdom	16
Italy	2
India	2
Russia	2
Spain	1
Bulgaria	1
Turkey	1

Four themes were identified from the student’s responses. These were: important positive interactions, a hostile learning environment, work life balance, and gender stereotypes. These themes are elaborated on below, supported alongside with quotes from the students.

### Important positive interactions

68 % (*n* = 17) of students shared positive experiences of their time. This positivity and optimistic perception of surgery was influenced by several factors including being involved in the team, uplifting role models, being taught basic surgical skills, and mentorship.

Students treasured moments where surgeons involved them, and friendly staff were welcoming. In theatre, they enjoyed it when surgeons explained the procedure, explained the anatomy, and did not ignore them. Being taught to suture and able to scrub into procedures inspired many students. It was noted that students enjoyed the practical aspect of surgery and being taught surgical skills excited them. Having a supportive and encouraging team positively impacted students. In particular, having an inspiring mentor boosted student’s perceptions of achieving a career in surgery.

### A hostile learning environment

A predominant theme of this study was students sharing their negative experiences regarding their time on surgical placements. Fifty-two percent of the students (*n* = 13) shared negative comments about their time on surgical placement, describing a learning environment which was not favorable.

This hostile learning environment seemed to be cultivated by numerous factors. Firstly, they felt unwelcomed, expressing feeling intimidated and being embarrassed. This culminated in an environment, which was not conducive to learning, and not inspiring for students.

### Work-life balance

Forty four percent of students (*n* = 11) shared concerns regarding work-life balance. This theme highlights student’s opinions and deliberations of the work-life balance of a career in surgery. After spending time in surgical rotations in medical school, they verbalized concerns about balancing a surgical career with having a family, spending time with loved ones, and having free time for hobbies and passions. Students seemed to have these opinions after witnessing the dynamics of doctors on placement and speaking to surgeons. Students were concerned about the competitive nature and difficulty of securing a job in surgery, and how this ultimately would negatively impact their work-life balance.

## Conclusion

Our study analyzed students’ experiences of surgical placements at university and their thoughts on a future career in surgery. We have highlighted how a hostile learning environment can have detrimental consequences on learning and greatly influence future career choice, and conversely, how positive impactful interactions can encourage learning and inspire career goals. Students are concerned about work-life balance; many feel that a surgical career cannot offer a healthy balance.

## Human ethics declaration

This research has been conducted in accordance with the 1964 Declaration of Helsinki.

## Consent to participate declaration

Each Participant gave oral and written informed consent to participate in this research.

## Declaration of competing interest

Eloise Owen; Jack Bennett; Chetan Parmar and Sjaak Pouwels have nothing to disclose.
